# Outcome of life-threatening arrhythmias among patients presenting in an emergency setting at a tertiary hospital in Accra-Ghana

**DOI:** 10.1186/s12872-022-02803-6

**Published:** 2022-08-07

**Authors:** Alfred Doku, Bernard Yeboah-Asiamah Asare, Richard Osei, Christian Owoo, Robert Djagbletey, Joseph Akamah, Ernest Aniteye, Dzifa Ahadzi

**Affiliations:** 1 Department of Medicine, University of Ghana Medical School, Korle-Bu Teaching Hospital, Accra, Ghana; 2 National Cardiothoracic Centre, Korle-Bu Teaching Hospital, Accra, Ghana; 3 Curtin School of Population Health, Curtin University, Perth, WA Australia; 4 Institute of Applied Health Sciences, University of Aberdeen, Aberdeen, UK; 5 Department of Anaesthesia, University of Ghana Medical School, Korle-Bu Teaching Hospital, Accra, Ghana; 6 Department of Anaesthesia, Korle-Bu Teaching Hospital, Accra, Ghana; 7 Department of Medicine, Korle-Bu Teaching Hospital, Accra, Ghana; 8 Department of Medicine, Tamale Teaching Hospital, Tamale, Ghana

**Keywords:** Arrhythmia, Bradycardia, Tachycardia, Management, Emergency, Ghana

## Abstract

**Background:**

Management of life-threatening arrhythmia can be incredibly challenging in advanced health systems. In sub-Saharan Africa (SSA), this is likely more challenging because of weak pre-hospital and in-hospital critical care systems. Little is known about life-threatening arrhythmia and their outcomes in SSA. The aim of this study was to examine the types and outcomes of arrhythmias among haemodynamically unstable patients presenting at a tertiary hospital in Accra-Ghana.

**Method:**

This was a retrospective case series study conducted at the Korle-Bu Teaching Hospital (KBTH), Accra-Ghana. Medical records of patients who presented with or developed haemodynamically unstable arrhythmias within 24h of admission from January 2018 to December 2020 were reviewed. The demographic characteristics and clinical data including outcomes of patients were collected. Descriptive statistics were used and results presented in frequency tables.

**Results:**

A total of 42 patients with life-threatening arrhythmias were included. Haemodynamically unstable tachyarrhythmias were the most common arrhythmias found among the patients (66.7%). Approximately 52% of patients had structural heart diseases whereas 26.2% had no apparent underlying cause or predisposing factor. Cardioversion (52.4%), commonly electrical (63.6%), and transvenous pacemaker implantation (23.8%) were the common initial interventions. The majority of the patients (88.1%) survived and were discharged home.

**Conclusion:**

Tachyarrhythmias are the most common haemodynamically unstable arrhythmias seen among patients presenting emergently in a leading tertiary hospital in Ghana. A high survival rate was observed and cannot be extrapolated to other healthcare settings in sub-Saharan Africa with limited resources to manage these clinical entities.

## Background

Arrhythmias refer to the rate, rhythm and conduction disturbances of the heart [1], which may occur as bradyarrhythmia (characterized by an abnormally slow heart beat with or without irregularity ) or tachyarrhythmia (characterized by an abnormally fast heart beat with or without irregularity) [2]. These disturbances could be caused by a range of underlying conditions including electrolyte abnormalities particularly hyperkalemia, endocrine disorders, structural heart diseases, respiratory conditions, drugs and post cardiac surgery [1]. Sudden onset of the arrhythmias could be an indication of systemic problems due to hypoxia, acid-base disturbances and multiple organ dysfunction [3]. Arrhythmias contribute significantly to hospital admissions, and are associated with high morbidity and mortality [4]. In Europe and North America, ventricular tachycardia (VT) or ventricular fibrillation (VF) is noted to account for almost 50% of sudden cardiac deaths [5]. Sustained VT and atrial fibrillation (AF) have been indicated to be the leading arrhythmias [6]. Arrhythmias are common conditions in critically ill patients [7], and in emergency departments (ED), the most common arrhythmia is AF [8]. A systematic review has documented a prevalence of 16–22% of Atrial fibrillation/flutter in heart failure and 3–7% in cardiology admissions in sub-Saharan Africa [9].

The clinical presentation, 12-lead surface electrocardiogram and response to vagal manoeuvres or drugs are useful in diagnosing the underlying causes of the arrhythmia and in determining the appropriate management, particularly in the emergency settings [10]. However, there is, to a large extent, limited data on the emergency presentation of arrhythmias in sub-Saharan Africa [9]. The management and prognosis of patients depend on well-staffed emergency rooms and critical care units, both of which are poorly developed in sub-Saharan Africa [11, 12]. We report on patients presenting with haemodynamically unstable arrhythmias at the Emergency Department (ED) of a tertiary teaching hospital in Ghana.

## Methods

### Study design and setting

This study is a retrospective case series study carried out at the Korle-Bu Teaching Hospital (KBTH), Accra-Ghana. KBTH is the largest and leading national referral and teaching hospital in Ghana, with a bed capacity of 2000 and about 1500 out-patient department visits every day.

### Patient population and data collection

All patients who presented with or developed haemodynamically unstable arrhythmias within 24 h whilst on admission were included in the study. Patients presenting between January 2018 and December 2020 at the emergency departments (EDs) of the KBTH including the general EDs and those of the Internal Medicine department and the National Cardiothoracic Centre were recruited. Haemodynamic instability was defined as blood pressure less than 90/60 mmHg with associated signs of tissue hypoperfusion or shock. Patients information included onset of admission until a clinical outcome. Clinical outcome was defined as either a discharge or death.

Using a structured extraction form, two resident physicians (trainee internist) collected data on the age and sex, clinical presentations, underlying predisposing factor, therapy received and outcomes from the medical records of patients. Clinical examinations findings by physicians and the cardiologists at the hospital were also captured. Electrocardiograph (ECG) and echocardiogram were done for all the patients, and coronary angiogram was carried out among some of those suspected of having coronary heart disease. Arrhythmic events were diagnosed based on the clinical features and a confirmatory ECG finding. The ECGs were performed at either the referring hospitals or at the KBTH. ECG interpretations captured were those done by either physician specialists or cardiologists. The ECGs were also reviewed by the study research team. Arrhythmia classification was done by analysing the ECG for arrhythmic events, and their occurrences were classified as either bradyarrhythmias or tachyarrhythmias.

Permission to use the clinical data was obtained from the Ethical and Protocol Review Committee (EPRC) of the KBTH.

### Data analysis

Data collected was entered into and analysed using Statistical Package for Social Sciences (SPSS) version 22.0. Categorical data was presented as frequencies and percentages. Continuous variables were presented as means and standard deviations.

## Results

### Baseline characteristics

A total of 42 patients aged 17 to 89 years (mean age 57.2 ± 20.9) were included in the study representing 0.114% or 114 in 100,000 patients per year admitted at the emergency department. Majority of the patients were males (54.8%). Pre-syncope (35.7%), palpitations (28.6%) and syncope (28.6%) were the most common symptoms among patients (Table 1). Seven patients presented with cardiac arrest and loss of consciousness and hence Triage Score of 1(critical). The rest were haemodynamically unstable but not critical and hence classified Triage Score of 2 (urgent).

**Table 1:** Clinical presentations of the patients

Characteristics	Frequency (%)
Age (years)	57.2 ± 20.9
Proportion of males	23 (54.8)
Presentation*	
Palpitations	12 (28.6)
Syncope	12 (28.6)
Pre-syncope^¥^	15 (35.7)
Chest pain	6 (14.3)
Dyspnea	6 (14.3)
Heart failure	5 (11.9)
Loss of consciousness	5 (11.9)
Cardiac arrest	2 (4.8)

*One patient could have more than one presentation

^¥^Pre-syncope: feeling of passing out or giddiness but without actual loss of consciousness

### Arrhythmias among patients and their underlining pathologies

Tachyarrhythmias were the most common type of arrhythmias observed among the patients (66.7%). Common tachyarrhythmias observed included AF (21.4%), supraventricular tachycardia (26.2%), which were mainly atrioventricular re-entry tachycardia (AVRT) and atrioventricular nodal re-entry tachycardia (AVNRT), and VT (14.3%). Heart blocks (28.6%), of which two-thirds (83.0%) was complete heart block, was the commonest bradyarrhythmia observed. One of the patients presented with brady-tachy syndrome or sick sinus syndrome (Table 2).

**Table 2:** Arrhythmia and structural observed among patients

Arrhythmias	Frequency (%)
Bradyarrhythmia	13 (30.9)
Tachyarrhythmia	28 (66.7)
Tachy-brady (sick sinus) syndrome	1 (2.4)
*Specific arrhythmias*	
Heart block	12 (28.6)
* Mobitz II AV-Block*	*2 (17.0)*
* Complete AV-Block*	*10 (83.0)*
Fast atrial fibrillation	9 (21.4)
* Atrial fibrillation with other arrhythmias*	*5 (55.6)*
* Atrial fibrillation*	*4 (44.4)*
Fast atrial flutter	2 (4.8)
Supraventricular tachycardia (AVRT and AVNRT)	11 (26.2)
Ventricular tachycardia	6 (14.3)
Ventricular fibrillation	2 (4.8)
*Underlying pathologies*	
Thyroid disease	1 (2.4)
Structural heart disease	22 (52.4)
* Hypertensive heart disease*	*9 (40.9)*
* Ischemic heart disease*	*6 (27.3)*
* Dilated cardiomyopathy*	*4 (18.2)*
*Valvular heart disease*	*2 (9.1)*
* Congenital heart disease (ASD)*	*1 (4.5)*
No identifiable cause	11 (26.2)
Others	8 (19.0)

Structural heart diseases (52.4%) were found to be the major underlying pathology of arrhythmias among the patients, with hypertensive heart disease (40.9%), ischaemic heart disease (27.3%) and dilated cardiomyopathy (18.2%) being the common types of structural heart diseases reported among the patients. Other causes identified included Bundle of Kent (Wolff Parkinson White Syndrome), respiratory acidosis secondary to upper airway obstruction, iatrogenic from atenolol use, hyperkalaemia, pulmonary embolism, stroke, diabetic ketoacidosis and congenital long QT syndrome. However, approximately 26% of the patients had no known or identifiable causes of arrhythmias.

### Management of arrhythmia

Cardioversion (52.4%)-commonly electrical cardioversion (63.6%), and pacemaker implantation (23.8%) were the common acute therapies offered to terminate and treat the arrhythmias respectively. For long term therapy, anti-arrhythmic drugs (40.5%)-commonly amiodarone (76.5%), permanent transvenous pacemaker implantation (9.5%) and percutaneous coronary intervention (7.1%) were common therapies (Table 3).

The line of management was typically based on the underlining pathology. In the short term, atrial fibrillation cases were managed with electrical cardioversion (44.4%), beta-blocker (22.2%), chemical cardioversion (Amiodarone) (11.1%), CPR (11.1%), and 1 case terminated spontaneously. Atrial flutter cases were managed using chemical cardioversion (Amiodarone) (50.0%) and electrical cardioversion (50.0%) in the short term. Supraventricular tachycardia cases were in the short term managed using electrical cardioversion (36.4%), chemical cardioversion (36.4%), defibrillation (18.2%) and beta-blocker (9.1%). Ventricular tachycardia cases were managed in the short term using electrical cardioversion (83.3%), chemical cardioversion (33.3%) which failed and 1 spontaneously terminated. Ventricular fibrillation cases were managed with CPR and defibrillation (i.e. cardioversion without synchronization) at the short term. For the AV- blocks, some were managed with CPR (16.7%), transvenous pacemakers (75%) and other who had acute reversible cause such hyperkalaemia were manage with administration of calcium gluconate, bicarbonate and insulin (25%).

**Table 3:** Therapies instituted for the management of Arrhythmia (*n* = 42)

Therapy*	Frequency (%)
Spontaneous termination (without therapy)	2 (4.8)
*Acute therapy*	
Valsalva Manoeuvre	2 (4.8)
Cardioversion	22 (52.4)
* Chemical (antiarrhythmic drugs)*	*8 (36.4)*
* Electrical*	*14 (63.60)*
Defibrillation	3 (4.8)
Beta-blocker	4 (9.5)
Transvenous pacemaker implantation	10 (23.8)
CPR (for cardiac arrest)	3 (7.1)
Hyperkalaemia correction	3 (7.1)
*Long term therapy*	
Antiarrhythmic drugs	17 (40.5)
* Amiodarone*	*13 (76.5)*
* Flecainide*	*1 (5.9)*
* Beta-blocker*	*3 (17.6)*
Mitral valve repair/replacement	1 (2.4)
Transvenous pacemaker implantation	4 (9.5)
Percutaneous coronary intervention	3 (7.1)
ICD implantation	1 (2.4)
ASD closure	1 (2.4)
Radiofrequency ablation	2 (2.4)
Heart failure management	2 (2.4)
Haemodialysis	1 (2.4)
Others	10 (21.4)
*One patient may be given more than one therapies during management of condition; patient may also receive a short-term and not be given a long-term therapy CPR = cardiopulmonary resuscitation; ICD = implantable cardioverter defibrillator; ASD = atrial septal defect	

### Clinical outcomes

Majority of patients (88.1%) were successfully treated and discharged. Five of the patients died, representing a mortality rate of 11.9%. One died from repeated arrhythmias, two from cardiac arrest and one from a thyroid storm. Among those who died, two presented with atrial fibrillation with very fast ventricular response which did not improve with electrical cardioversion and antiarrhythmic medication and hence died from cardiac arrest. Another two presented with complete heart block from hyperkalaemia and managed with insulin, calcium gluconate and sodium bicarbonate and CPR as the short-term therapy but died before we could arrange for dialysis; and one presented with supraventricular tachycardia from thyroid storm but did not response to acute therapy.

## Discussion

In this study, we report on the presentation of life-threatening arrhythmias among patients at the ED of the KBTH, Ghana. It was found that pre-syncope, palpitations and syncope were the common presenting complaints. Tachyarrhythmia was the prevalent arrhythmia. Arrhythmias were mostly caused by structural heart diseases and arrhythmias were terminated mostly using cardioversion in the acute phase and the long-term with the use of anti-arrhythmic drugs.

Symptoms particularly syncope, palpitations and light-headedness caused by cardiac arrhythmias are indicated to be the common complaints among patients presenting at EDs and could be responsible for 3 to 4% of all ED visits [11]. This confirms the observation made in our study where syncope, pre-syncope and palpitations were the commonest presenting complaints. It has been estimated that approximately 90% of patients presenting with syncope of cardiac origin have ECG abnormalities [12].  It has been indicated  that a clinical presentation of palpitations together with syncope, dizziness or near syncope most often suggests a life-threatening tachyarrhythmia [13].

Our current study found tachyarrhythmias as the most common arrhythmia. This finding agrees with what has been found among critically ill patients. In a study by Reinelt et al. [14], out of 310 episodes of arrhythmias observed, 278(89.7%) were tachyarrhythmias and 32(10.3%) bradyarrhythmias. In contrast, an international meta-analysis reported bradyarrhythmias (4.8%) as the most frequent life-threatening arrhythmias compared to tachyarrhythmias (2.6%) among patients presenting at an ED with syncope [15]. The observed difference could be due to differences in the study designs used.

Heart blocks, AF, supraventricular tachycardia (AVRT and AVNRT) and VT were found as the most common haemodynamically unstable arrhythmias. AF has been indicated to be the commonest arrhythmia observed in EDs [8]. In a population-based study over an 8-year period, AF accounted for 0.5% of all ED visits [16]. This conforms, in part, to the finding of our study where AF was the most common tachyarrhythmia observed. In the current study, about 56% of AF was observed together with other arrhythmias including atrial flutter, VT, VF and torsades de pointes. This was observed in five of the patients at different times during the admission.

Supraventricular tachycardia seen in our study is similar to the finding of the study by Brunetti et al. [17], which showed that junctional supraventricular arrhythmias (AVNRT) were the most common arrhythmias. Sustained VT is not common as it has been indicated to be responsible for only 0.05% of patients visiting the ED, however unsustained VT is a common finding with an incidence of 2 to 7% among patients in critical care settings [18].

In this current study, AV block was found to be the most frequent bradyarrhythmia and the commonest arrhythmias seen in 12 patients (28.6%); Mobitz II AV-block, and complete heart block. Significant AV conduction disturbances including second-degree type 2 AV-block, advanced AV-block, third-degree AV-block with junctional escape rhythm, ventricular escape rhythm have also been found among emergency medical service patients referred for syncope [17].

One patient in the study had an episode of sick sinus or brady-tachy syndrome requiring permanent pacemaker implantation and beta-blocker therapy. A similar observation was also made among patients requiring a permanent pacemaker in another study [19].

The current study found structural heart diseases as the most common underlying pathology for arrhythmias. In a meta-analysis study, patients with left ventricular hypertrophy were found to be at higher odds of developing supraventricular arrhythmias [20]. Downey et al. has also reported ischemic heart disease as more common in patients with arrhythmias [21]. In our study, congenital heart disease accounted for arrhythmia in one of the patients. In adults, it has been stated that arrhythmias are the frequent long-term complication of congenital heart disease [22]. In a study among patients with congenital heart disease, arrhythmias occurred in 32% of them after undergoing surgery [23]. Arrhythmia in one of the patients in our study was found to be caused by valvular heart disease particularly mitral valve prolapse. It has also been reported that mitral valve prolapse may cause ventricular arrhythmia and is an under-estimated cause of arrhythmic sudden cardiac death, particularly in young adults [24]. Dilated cardiomyopathy was found to cause arrhythmia in four (9.5%) of the patients in our study. A study has also identified arrhythmias in 18.7% of patients with peripartum cardiomyopathy, a type of dilated cardiomyopathy [25]. However, 26.2% of the patients in the current study had no known underlying pathology for arrhythmias. This is comparable to findings established in patients with AF, the most frequent arrhythmia, where the cause of up to 30% of cases may not be known [26].

On the management of arrhythmias, the study found that cardioversion mostly electric cardioversion as an acute therapy was carried out in 14 of the patients (33.3%) to terminate arrhythmias on account of the hemodynamic instability. Cardioversion either chemical or electrical is employed to re-establish normal sinus rhythm to ease symptoms and restore sinus rhythm [27]. In the acute treatment of cardiac arrhythmias, it has been indicated that direct current transthoracic cardioversion is performed to re-establish normal sinus rhythm in haemodynamically unstable patients [27]. This could justify the observation made in our study. It was observed in the study that three of the patients were defibrillated, with one of them as many as 53 times. Hood et al. have stipulated that repeating unsuccessful cardioversion with amplified power, varying the vector, use of paddles with manual compression, or a few permutations is frequently successful [27]. However, it has been recommended particularly in the acute treatment of AF that electrical cardioversion may be done in ED with 150–200 biphasic Joules as the first energy setting to avoid repeated shocks and VF that could arise from uncoordinated cardioversion of AF with lower voltages [8]. However, in our study, one patient with AF was electrically cardioverted with 90 Joules whereas in 2 of the patients with VF electric cardioversion with 120 joules with primary success.

A Transvenous pacemaker was the second common acute therapy performed in our study, primarily for bradyarrhythmias. In patients presenting with hemodynamically unstable bradycardia, pacing the heart either transvenously or transthoracically is recommended [27]. This is in line with what was seen in our study.

A high survival rate was observed in our study. This could be attributed to the fact that the study centre, Korle-Bu Teaching Hospital, is the leading teaching (tertiary) hospital in Ghana, and may have the medical equipment, skills and knowledge of staff to manage the cases. This finding cannot be extrapolated to other healthcare facilities and settings in sub-Saharan Africa with limited resources such as “cardiac implantable electronic devices insertions” and “invasive arrhythmia treatment centres” (p1) [9] to manage these clinical entities.

### Limitations

Limitations to the study were noted.

The study relied mainly on the medical records of patients, which may have recordkeeping mistakes such as wrong input of age, and uncertainty in the data recorded such as arising from situations where differential diagnoses are initially noted at admission, but final diagnoses not recorded [28]. However, the research team reviewed all clinical and laboratory/imaging findings of each patient to confirm the diagnoses.

The study findings are limited to haemodynamically unstable arrhythmias patients and conducted at a single health facility (setting) in the Greater Accra region of Ghana, though the national referral centre, limiting the generalizability of the study findings to other healthcare facilities and settings in Ghana and sub-Saharan Africa. However, the findings of this study could be indicative of the condition considered at other similar settings in Ghana and sub-Saharan Africa.

## Conclusion

The incidence of haemodynamically unstable acute arrhythmias at the ED of the KBTH has a case fatality rate of 11.9%. Pre-syncope, palpitations and syncope were the common presenting complaints of patients presenting with haemodynamically unstable arrhythmias. Tachyarrhythmia was the most common arrhythmia among patients. Arrhythmias were mostly caused by structural heart diseases however, in a significant number of patients identifying an underlying cause or predisposing factor was elusive. Patients were managed mostly by cardioversion or pacemaker implantation in the acute phase and long term using anti-arrhythmic medication. Interventions should be readily made available in all referral hospitals for the successful management of emergency arrhythmias.

## Data Availability

The datasets during and/or analysed during the current study available from the corresponding author upon reasonable request.

## Abbreviations

AF: Atrial fibrillation
AV: Atrioventricular
AVRT: Atrioventricular re-entry tachycardia
AVNRT: Atrioventricular nodal re-entry tachycardia
ASD: Atrial septal defect
ECG: Electrocardiograph
ED: Emergency department
ICD: Implantable cardiac defibrillator
KBTH: Korle-Bu teaching hospital
VT: Ventricular tachycardia
VF: Ventricular fibrillation
